# Further Steps Toward the Development of Gluten Reference Materials – Wheat Flours or Protein Isolates?

**DOI:** 10.3389/fpls.2020.00906

**Published:** 2020-07-07

**Authors:** Eszter Schall, Katharina A. Scherf, Zsuzsanna Bugyi, Kitti Török, Peter Koehler, Regine Schoenlechner, Sándor Tömösközi

**Affiliations:** ^1^Research Group of Cereal Science and Food Quality, Department of Applied Biotechnology and Food Science, Budapest University of Technology and Economics, Budapest, Hungary; ^2^Leibniz-Institute for Food Systems Biology at the Technical University of Munich, Freising, Germany; ^3^Department of Bioactive and Functional Food Chemistry, Institute of Applied Biosciences, Karlsruhe Institute of Technology (KIT), Karlsruhe, Germany; ^4^Biotask AG, Esslingen am Neckar, Germany; ^5^Department of Food Science and Technology, University of Natural Resources and Life Sciences, Vienna, Austria

**Keywords:** celiac disease, gliadin, gluten, reference material, ELISA, wheat flour, protein isolates

## Abstract

Celiac disease is a gluten-induced hypersensitivity reaction that requires a lifelong gluten-free diet. Gluten-free foods must not contain more than 20 mg/kg gluten as laid down by Codex Alimentarius. Measuring the presence of gluten with routine immunoanalytical methods in food is a serious challenge as many factors affect accurate determination. Comparability of the results obtained with different methods and method validation are hindered by the lack of a widely accepted reference material (RM). The core questions of RM development from wheat are the number of cultivars to be included and the format of gluten (i.e., flour, gluten, or gliadin isolates) to be applied. Therefore, the aim of our work was to produce an appropriate gluten RM from wheat. For this, five previously selected wheat cultivars and their blend were used to produce flours, gluten and gliadin isolates under laboratory conditions. Protein content, protein composition and responses to different ELISA methods were compared and widely evaluated in our study. The protein contents of the flours were 12.1–18.7%, those of the gluten isolates 93.8–97.4% and those of the gliadin isolates 72.7–101.9%. The gluten and gliadin isolates had similar protein profiles as the source flours. By comparing the different wheat cultivars and their protein isolates, we found that the isolation had a smaller effect on protein composition than genetic variability. The choice of a blend would be more suitable for the production of a RM in case of flours and also isolates. The immunoanalytical results showed that the isolation had an effect on the analytical results, but its extent depended on the ELISA method. The use of flour would be more applicable in this regard, but handling of the material and long-term stability should also be considered in the final decision of gluten RM production.

## Introduction

Wheat, rye, and barley are widely used cereals in the food industry because of their nutritional quality and beneficial technological properties (Shewry and Tatham, 2016). However, their consumption can cause health problems for some people. One of the most common cereal-induced hypersensitivity reactions is celiac disease, which is a disorder with an autoimmune component associated with serious damage of the small intestinal mucosa. The triggers of celiac disease are the storage proteins of gluten-containing cereals (Leonard et al., 2017). Since patients can only be treated with a gluten-free diet, the availability of gluten-free foods is essential. According to the Codex Alimentarius, foods can be labeled gluten-free if the gluten content does not exceed 20 mg/kg (Codex Stan 118-1979,2015). Thus, methods for the reliable quantitation of gluten in (gluten-free) foods are needed. One of the analytical problems is that gluten is not a homogeneous, properly defined component, but a mixture of heterogeneous proteins with different physico-chemical properties (Tatham and Shewry, 2012). According to the classical Osborne fractionation, gluten proteins from wheat, rye, and barley can be divided into alcohol-soluble prolamins and glutelins which are not soluble in aqueous alcohol solutions (Osborne, 1907; Koehler and Wieser, 2013). Cereal prolamins have trivial names: gliadins for wheat, secalins for rye and hordeins for barley. However, only wheat glutelin has a trivial name, which is glutenin (Wieser and Koehler, 2009; Koehler and Wieser, 2013). Cereal proteins can be further classified based on their size and electrophoretic mobility. Wheat gluten proteins are subdivided into α-/β-gliadins (QPQPF), γ-gliadins (QQPQQPFP), ω1,2-gliadins (QPQQPFP), and ω5-gliadins (QQQPF), low-molecular-weight glutenin subunits (LMW-GS) (QQPPFS) and high-molecular-weight glutenin subunits (HMW-GS) (QQPGQG, YYPTSP) (Koehler and Wieser, 2013). The typical repetitive amino acid sequences (epitopes) of wheat gluten proteins, examples of which are given in parentheses, are involved in the induction of celiac disease. These sequences have high contents of proline and glutamine, which make them resistant to protein-degrading digestive enzymes (Brouns et al., 2019). Most of the reactive epitopes have been reported in the gliadin fraction and, e.g., α-gliadin contains a peptide with a length of 33 amino acids that was shown to be highly celiac disease-active (Shan et al., 2002). Further studies have shown that other gluten protein types also contain celiac disease-active epitopes (Lexhaller et al., 2019; Sollid et al., 2020).

Several analytical methods based on different mechanisms are available for gluten quantitation (Scherf and Poms, 2016). DNA-based techniques, such as polymerase chain reaction (PCR), sensitively detect DNA segments coding for gluten proteins, but have the disadvantage that gluten proteins are not directly determined (Mujico et al., 2011; Codex Stan 118-1979,2015). The number of studies using liquid chromatography mass spectrometry (LC-MS) for gluten quantitation has increased, as they are capable of determining all gluten protein types from gluten-containing cereals. However, routine application of LC-MS is limited because of the high level of expertise required and the cost of instrumentation (Schalk et al., 2018). The most common method used in routine analysis is the enzyme-linked immunosorbent assay (ELISA) based on the immunochemical reaction between epitope(s) within gluten proteins and an epitope-specific antibody. The advantages of the method are its relatively easy implementation and the specific and sensitive detection (Diaz-Amigo and Popping, 2013; Bruins Slot et al., 2016). The different ELISA methods available on the market offer various solutions for sample preparation, test format (sandwich or competitive), type of antibody (monoclonal or polyclonal), specificity of the antibody toward different epitopes and calibration material (Lexhaller et al., 2016; Scherf and Poms, 2016). This is why several studies have shown that different ELISA kits give different results when the same samples are analyzed (Bugyi et al., 2013; Rzychon et al., 2017; Scherf, 2017).

The accurate determination of the gluten content of food is a challenge because the identification of factors affecting the analytical results is difficult. One step toward harmonization of analytical methods will be the availability of a universally accepted gluten reference material (RM) (Poms, 2006).

According to the ISO guide 30, a RM is a material that is sufficiently homogenous and stable with respect to one or more specific properties, which has been established to be fit for its intended use in a measurement. Its production must be reproducible, and it should be easy to handle (Diaz-Amigo and Popping, 2013; Scherf and Poms, 2016). Further, a certified RM is a RM that is characterized by a metrologically valid procedure for one or more specified properties, accompanied by a certificate providing the value of the specified property, its associated uncertainty, and a statement of metrological traceability. A certified RM would provide an opportunity to support method validation and to identify the factors influencing gluten analysis. The most widely used standard-like material in gluten analysis is a gliadin isolate called Prolamin Working Group (PWG)-gliadin (van Eckert et al., 2006; van Eckert et al., 2010). The advantage of the material is its high purity, good solubility and detailed characterization. PWG-gliadin was proposed for approval as a certified RM, but it did not meet some of the RM requirements for certification, such as reproducibility of production (Diaz-Amigo and Popping, 2013). Consequently, there is still a need for a gluten RM, but there are a number of questions about its composition (one cultivar or a blend for each species of wheat, rye, and barley) and type (flour or protein isolate) that need to be investigated and answered. In order to provide solid foundations for a comprehensive RM for gluten from wheat, rye, and barley, we started our investigations with wheat, because it is by far the most widely used species of the three. All learnings from our studies on wheat will enable us to easily and efficiently transfer these to the development of RM for rye and barley, because a universal gluten RM should certainly include the relevant proteins of all three species.

Within an international cooperation, the factors affecting gluten analysis (such as genetic and environmental variability) were investigated with the aim to design a gluten RM candidate. For this purpose, 23 wheat cultivars collected from different geographical locations around the world were examined and characterized in detail (Hajas et al., 2018). Based on the results of this study, five cultivars were selected and investigated for the magnitude of the analytical error of ELISA methods resulting from the use of one cultivar or their blend (Schall et al., 2020). Another major issue of RM production is the decision whether to use flour, a gluten isolate or a gliadin isolate. The flour represents gluten contamination most realistically and its production is relatively simple, but it contains components (e.g., lipids) causing instability during storage (Wang and Flores, 1999). The storage stability and handling of the isolates could be more advantageous, but the protein composition may change during isolation which could affect the analytical results (Diaz-Amigo and Popping, 2012). The gliadin isolate has the advantage of being completely soluble in specific solvents, but it does not contain all protein types that induce celiac disease.

In this work we investigated the effect that the production of protein isolates (gluten or gliadin) from wheat flour has on the amount and composition of proteins compared to the flour. Furthermore, the suitability of the RM material candidates for different analytical methods for gluten quantitation were evaluated to enable the selection of a proper RM. By examining the blend of the five wheat cultivars, the use of individual cultivars and their blend not just as flour but also as protein isolates was possible.

## Materials and Methods

### Wheat Samples

Five wheat (*Triticum aestivum* L.) cultivars were selected in line with a set of selection criteria described in our previous study (Hajas et al., 2018) and collected from the harvest year of 2016 for this work: Akteur (Germany); Carberry (Canada); Mv Magvas (Hungary); Yitpi (Australia), and Yumai-34 (China).

### Production of Wheat Flours

The moisture content of the grains was determined by an InfratecTM 1241 Grain Analyzer (Foss Tecator AB, Höganäs, Sweden). The wheat samples were conditioned prior to milling according to Hungarian Standard MSZ 6367-9:1989,1989. The tempered kernels were milled on a laboratory mill (FQC 109, Metefém, Budapest, Hungary). The whole-meal was sieved on a 250 μm sieve for 20 min (AS 200 basic, Retsch GmbH, Haan, Germany). The blend of the five cultivars was prepared by mixing equal amounts (80 g each) of grains from the single cultivars by shaking in a closed container manually for 10 min before milling. The homogeneity of the blend was confirmed later by chemical composition data in section “Comparison of the Different Gluten and Gliadin Isolates From Individual Cultivars and Their Blend.”

### Production of Gluten and Gliadin Isolates

Gluten and gliadin isolates were prepared based on the standard for wet gluten production and the study reporting the production of PWG-gliadin (AACC Method 38-12.02,2000; van Eckert et al., 2006). The Glutomatic System (Perten Instruments, Hägersten, Sweden) was used for the removal of albumins and globulins with 0.4 M NaCl solution from white flours of each cultivar and the blend. The resulting gluten was further washed for another 10 min with tap water to remove residual starch and salt. The gluten was freeze-dried for 24 h (Christ Alpha 1-4 LOC-1M, Martin Christ Gefriertrocknungsanlagen GmbH, Osterode am Harz, Germany). Then the dry gluten was ground with knife mills for 3 × 10 s at 7000 rpm (Grindomix GM200, RETSCH GmbH, Haan, Germany). One-third of the total amount (ca. 10 g) of isolated gluten was collected separately for protein content and composition analysis. The gliadins were then extracted three times with 270 mL 60% (v/v) ethanol from the two-thirds of the total amount of dry gluten. The suspension was stirred for 30 min with a magnetic stirrer followed by centrifugation for 15 min at 4500 *g* (Labofuge 400R, Heraeus, Kendro Laboratory Products, Germany). The supernatants were combined and freeze-dried. In order to verify the reproducibility of the production, two independent batches of samples were prepared from milling to isolation on the arbitrarily selected Akteur cultivar.

### Determination of Crude Protein Content

The nitrogen content of the flours and the isolates was determined by a Leco FP 528 nitrogen analyzer (Leco Corporation, St. Joseph, United States) in duplicates following the MSZ En Iso 16634-2:2016,2016. The nitrogen content was multiplied by 5.7 to obtain the crude protein content.

### Calculation of the Relative Amount of Isolates

The following calculations were used to determine the relative amount of materials obtained during the isolation:

Amount of gluten proteins relative to flour proteins (%):

$$\frac{a\,m\,o\,u\,n\,t\,o\,f\,g\,l\,u\,t\,e\,n\,p\,r\,o\,t\,e\,i\,n\,s\,e\,x\,t\,r\,a\,c\,t\,e\,d\,f\,r\,o\,m\,f\,l\,o\,u\,r\,\left(\frac{g}{100\,g}\right)}{p\,r\,o\,t\,e\,i\,n\,c\,o\,n\,t\,e\,n\,t\,o\,f\,f\,l\,o\,u\,r\,\left(\frac{g}{100\,g}\right)}\times 100$$

Amount of gliadin proteins relative to gluten proteins (%):

$$\frac{a\,m\,o\,u\,n\,t\,o\,f\,g\,l\,i\,a\,d\,i\,n\,p\,r\,o\,t\,e\,i\,n\,s\,e\,x\,t\,r\,a\,c\,t\,e\,d\,f\,r\,o\,m\,f\,l\,o\,u\,r\,\left(\frac{g}{100\,g}\right)}{a\,m\,o\,u\,n\,t\,o\,f\,g\,l\,u\,t\,e\,n\,p\,r\,o\,t\,e\,i\,n\,s\,e\,x\,t\,r\,a\,c\,t\,e\,d\,f\,r\,o\,m\,f\,l\,o\,u\,r\,\left(\frac{g}{100\,g}\right)}\times 100$$

### Protein Characterization by SE-HPLC

Protein extracts were prepared for size-exclusion high-performance liquid chromatography (SE-HPLC) analyses according to Batey et al. (1991) and Gupta et al. (1993) with minor modifications. Acetonitrile (50%, v/v) containing 0.1% (v/v) trifluoroacetic acid (TFA) was used as the extraction solvent. Wheat flour (15 mg)/gluten isolate (1.5 mg)/gliadin isolate (1.5 mg) was suspended in 1 mL of the extraction solvent and shaken (1,500 rpm, 30 min, 20–22°C) followed by centrifugation (4,500 × g, 20 min, 20°C). The supernatant was collected (extractable protein fraction). The remaining pellet was extracted with 1 mL of the same extraction solution using sonication for 40 s with an amplitude of 90%. Then, samples were shaken (1,500 × rpm, 30 min, 20–22°C) and centrifuged (4,500 *g*, 20 min, 20°C) to obtain a supernatant (unextractable protein fraction). All supernatants were filtered (Minisart^®^, 15/0.45 RC, Sartorius AG, Goettingen, Germany) before SE-HPLC analysis. The extractions were done in duplicate for each flour sample. The conditions for the SE-HPLC analyses were the following: instrument: PerkinElmer Series 200 HPLC with TotalChrom Navigator v6.2.1 (PerkinElmer Inc., Shelton, CT, United States); column: BioSep-SEC-s4000 (particle size 5 μm, pore size 50 nm, 300 × 7.8 mm, separation range for proteins 15,000–1,500,000, Phenomenex, Torrance, CA, United States); temperature: 25°C; injection volume: 20 μL; elution solvents: 50% (v/v) acetonitrile containing 0.1% (v/v) TFA; flow rate: 1 mL/min; running time: 20 min, detection: UV absorbance at 214 nm. After each run, the column was equilibrated with the elution solvent for 1 min. The chromatograms of the extractable and unextractable proteins were divided into three sections: the proportion of polymeric, monomeric and albumin/globulin fractions to “total extracted” protein were calculated from the peak areas as percentage of the total peak area.

### Protein Characterization by RP-HPLC

Wheat flours (100 mg) were extracted sequentially according to the modified Osborne procedure (Wieser et al., 1998) by magnetic stirring with salt solution (extraction of albumins/globulins), followed by 60% (v/v) ethanol solution (extraction of gliadins), and glutelin extraction solution [containing 1-propanol, tris(hydroxymethyl)aminomethane hydrochloride, dithiothreitol and urea for the extraction of glutenins]. All suspensions were centrifuged (3550 *g*, 25 min, 20°C) and the supernatant filtered (Whatman^TM^ Spartan 13/0.45 RC, GE Healthcare, Freiburg, Germany). The gluten isolates (20 mg) were extracted with 60% (v/v) ethanol solution and glutelin extraction solution in the same way as the flours. The gliadin isolates (5 mg) were extracted with 60% (v/v) ethanol solution in the same way as the flours. The extractions were done in triplicate for each sample. The conditions for reversed-phase high-performance liquid chromatography (RP-HPLC) analyses were the following: the instrument was Jasco XLC with Jasco Chrompass Chromatography Data System (Jasco, Pfungstadt, Germany); column: Acclaim^TM^ 300 C_18_ (particle size 3 mm, pore size 30 nm, 2.1 × 150 mm, Thermo Fisher Scientific, Braunschweig, Germany); temperature: 60°C; elution solvents: TFA (0.1%, v/v) in water (A) and TFA (0.1%, v/v) in acetonitrile (B); linear gradient: 0 min 0% B, 0.5 min 20% B, 7 min 60% B, 7.1–11 min 90% B, 11.1–17 min 0% B for albumins/globulins; 0 min 0% B, 0.5 min 24% B, 20 min 56% B, 20.1–24.1 min 90% B, 24.2–30 min 0% B for gliadins and glutenins; flow rate: 0.2 mL/min; injection volume: 20 mL for albumins/globulins and glutenins, 10 mL for gliadins; detection: UV absorbance at 210 nm. The protein contents of the extracts were calculated from the absorbance areas using 5, 10, 15, and 20 μL of a PWG-gliadin solution (2.5 mg/mL in 60% ethanol) (van Eckert et al., 2006) as calibration reference. The contents of ω5-, ω1,2-, α-, and γ-gliadins were calculated from the absorbance area of each gliadin type relative to the total gliadin content, as were those of glutenin-bound ω-gliadins (ωb-gliadins), HMW-GS and LMW-GS relative to the total glutenin content.

### Gliadin/Gluten Quantitation With ELISA Methods

The gliadin/gluten quantitation was performed with two commercially available ELISA test kits: the AgraQuant Gluten G12 Assay (COKAL0200, Romer Labs, Tulln, Austria) and the RIDASCREEN Gliadin Assay (R7001, R-Biopharm, Darmstadt, Germany). They apply different antibodies (monoclonal G12 and monoclonal R5, respectively) and are calibrated differently (vital wheat gluten extract and PWG-gliadin, respectively). ELISA procedures were carried out according to the kit instructions. Three independent extractions were performed for each sample. The absorbances were determined using a microplate reader (iMark^TM^ Microplate Absorbance Reader, Bio-Rad, Hercules, CA, United States). The gliadin/gluten concentration was calculated from the absorbance values by the Bio-Rad Microplate Manager 6 software (Bio-Rad, Hercules, CA, United States) using the curve fit suggested by the manufacturer. The ELISA test kits used for analysis were randomly coded with capital letters (A and B) in section “Results and Discussion.”

### Statistical Analysis

The analytical results were statistically evaluated with the investigation of means, standard deviations, one-sample *t*-test and analysis of variance (ANOVA) with Fisher’s Least Significant Difference (LSD) *post hoc* test at a confidence level of 0.95 using Statistica 13 software (TIBCO Software Inc., Palo Alto, CA, United States).

## Results and Discussion

### Investigation of the Reproducibility of Flour, Gluten, and Gliadin Production

An important aspect of choosing a proper RM is the reproducibility of its production. Therefore, we produced two independent batches from the cultivar “Akteur” in parallel with a method based on preliminary experiments and all protein parameters of each type of sample (flour – gluten isolate – gliadin isolate) were investigated. The crude protein content of the laboratory milled batch 1 and 2 flour was 14.6 and 13.9%, respectively (Table 1). The difference was significant, but this variation was smaller than the differences between the five cultivars used in this study. Gluten production can be affected by a number of factors that determine the final protein content and yield (Van Der Borght et al., 2005). Depending on the preparation, it may contain starch, lipids and fibers. The amount of starch varies, but with extensive washing a significant reduction of starch embedded in the protein matrix can be observed. However, starch and fiber become entrapped in the cohesive matrix of the protein and become more difficult to remove as the protein content increases (Saulnier et al., 1997; Day et al., 2006). The non-polar lipids of wheat flour interact with the hydrophobic regions of gluten proteins during the washing process, not allowing complete extraction of lipids (Day et al., 2006). A higher protein content could mean greater purity of the gluten isolate, but there may be small amounts of soluble proteins trapped in the gluten matrix as well (Ortolan and Steel, 2017). Therefore, protein content alone is not sufficient to determine gluten quality, which is why the protein profile of isolates should be examined. The crude protein content of the batch 1 gluten isolate was 96.6% while batch 2 had 97.1%, revealing that the isolates had high purity (Table 1). The amount of gluten proteins relative to the amount of proteins from flours by weight was 63% for batch 1 and 75.3% for batch 2 (Table 1). With our laboratory method, we were able to produce a gluten isolate with a high and constant protein content, but the extra manual washing step in our method could affect the amount of soluble proteins and starch within the gluten matrix and could also mean the loss of gluten proteins. In case of the production of our gliadin isolate, the non-protein components were probably less involved. However, the gluten proteins themselves form a complex system, which makes it difficult to produce a constant quality gliadin isolate. The crude protein content of the batch 1 gliadin isolate was 96.4% while it was only 76% for batch 2 which was a more substantial divergence than between the different batches of gluten isolates (Table 1). Interestingly, in both cases similar amounts of gliadin proteins were obtained from gluten isolates: 36.5% for batch 1 and 34.6% for batch 2 (Table 1). In the case of PWG-gliadin, the most widely used gliadin standard, a large amount of good quality material was produced, which was tested by several methods, but yield data are not available (van Eckert et al., 2006). Rallabhandi et al. (2015) produced prolamins including gliadin isolates in laboratory conditions. Their gliadin material contained 68% proteins with a yield of 1.44 g/100 g flour (Rallabhandi et al., 2015). The yield and protein content of gliadin isolates may also depend on the methods. Publications for the production of prolamins focus mainly on matching with the source flour, so comparing the protein profile of gliadin isolates with flours and gluten isolates is essential (van Eckert et al., 2006; Huang et al., 2017; Schalk et al., 2017).

**TABLE 1:** Crude protein content of flours, gluten and gliadin isolates; amount of gluten proteins obtained from flour proteins and amount of gliadin proteins obtained from gluten proteins (all values are expressed on dry matter basis).

Sample	Parameter				
	Crude protein content of flours (%)^abc^	Crude protein content of gluten isolates (%)^abc^	Amount of gluten proteins relative to flour proteins (%)	Crude protein content of gliadin isolates (%)^abc^	Amount of gliadin proteins relative to gluten proteins (%)
Akteur – batch 1	14.6^+^ ± 0.0	96.0 ± 0.4	63.0	96.4^+^ ± 0.2	36.5
Akteur – batch 2	13.9^D^ ± 0.0	97.1^A^ ± 0.3	75.3	76.0^E^ ± 1.0	34.6
Carberry	18.7^A^ ± 0.1	95.3^B^ ± 0.0	76.9	87.0^B^ ± 0.8	34.0
Mv Magvas	12.1^E^ ± 0.1	97.4^A^ ± 0.0	75.5	72.7^F^ ± 0.9	36.9
Yitpi	16.6^B^ ± 0.1	93.8^D^ ± 0.2	73.4	101.9^A^ ± 1.5	58.2
Yumai-34	16.7^B^ ± 0.0	94.5^C^ ± 0.3	76.8	83.3^C^ ± 1.6	52.8
Blend	15.4^C^ ± 0.1	95.5^B^ ± 0.1	78.5	79.6^D^ ± 1.5	28.8
Mean of the five cultivars	15.6	95.6	75.6	84.2	43.3
PWG-gliadin	–	–	–	92.8 ± 0.8	–

**^*a*^Within each column, the mean value of batch 1 Akteur samples marked with a plus sign is significantly different from the mean value of batch 2 Akteur samples (p < 0.05; one-way ANOVA). ^b^Within each column, the measured value of the blend marked with an asterisk is significantly different from the calculated mean of the five cultivars (p < 0.05; one-sample t-test). ^*c*^Within each column, mean values marked with different capital letters are significantly different (p < 0.05; factorial ANOVA, Fisher’s LSD).**

**FIGURE 1:**
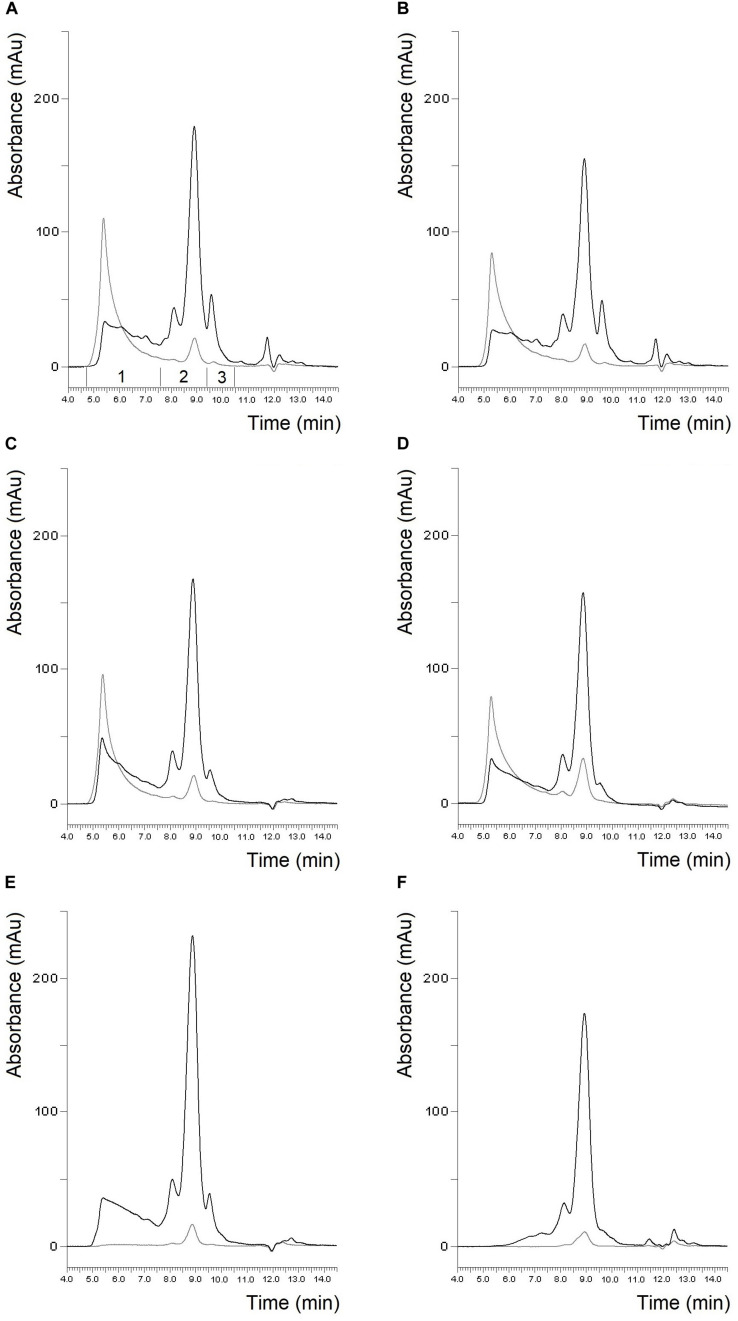
Protein profile of batch 1 flour **(A)**, batch 2 flour **(B)**, batch 1 gluten isolate **(C)**, batch 2 gluten isolate **(D)**, batch 1 gliadin isolate **(E)** and batch 2 gliadin isolate **(F)** from Akteur cultivar determined by SE-HPLC (black line: soluble fraction, gray line: insoluble fraction, 1: polymeric proteins; 2: monomeric proteins; 3: albumins/globulins).

The protein composition of the two batches of materials separated by SE-HPLC is shown in Figure 1. It can be clearly seen that the protein profiles of the two batches of flours were quite similar both in the soluble and insoluble protein fractions as in the case of gluten isolates. The only conspicuous difference was the higher albumin/globulin peak in the batch 1 gluten isolate. The similarity between the two batches both in flours and gluten isolates was also supported by the distribution of monomeric and polymeric proteins as there were no significant differences between the two batches (Table 2). In case of gliadin isolates, the protein profiles between the two batches in the SE-HPLC chromatograms showed differences, as the presence of polymer-like proteins was observed in batch 1 (Figure 1). The insoluble fractions of gliadin isolates – with the expectation of a small peak – did not show any higher molecular weight proteins. Glutenin proteins obtained during the isolation were also analyzed in each case (results are not shown), and the two glutenins produced in parallel were similar, which appeared mostly in the insoluble fraction. It is conceivable that the problem with those higher molecular weight proteins appearing in gliadins could be the poor solubility in the solvent used in the SE-HPLC method. The peaks typical for monomeric proteins (between 7.5 and 9.5 min) were similar in the two batches of gliadin isolates. A characteristic value of monomeric proteins could be the ratio of the two peaks appearing in the chromatograms of the soluble fraction, which was 4.98 for batch 1 and 5.92 for batch 2.

**TABLE 2:** Ratio of monomeric and polymeric protein fractions of flours, gluten and gliadin isolates determined by SE-HPLC (monomeric and polymeric protein contents are expressed as percentage of total monomeric and polymeric extract; all values are expressed on dry matter basis).

Sample		Parameter			
		Monomeric (%)^abc^	Polymeric (%)^abc^	Monomeric/Polymeric ratio	Monomeric Peak 2/Peak 1 ratio
Flour	Akteur – batch 1	47.8 ± 1.9	52.2 ± 2.1	0.92	4.14
	Akteur – batch 2	46.9^F^ ± 1.9	53.1^A^± 2.1	0.88	4.29
	Carberry	51.5^BCDE^ ± 2.1	48.5^BCD^± 1.9	1.06	2.68
	Mv Magvas	49.2^EF^ ± 2.0	50.8^AB^± 2.0	0.97	5.26
	Yitpi	49.9^DEF^ ± 2.0	50.1^ABC^± 2.0	1.00	4.02
	Yumai-34	51.8^BCDE^ ± 2.1	48.2^BCD^± 1.9	1.07	4.36
	Blend	50.5^CDE^ ± 2.0	49.5^BC^± 2.0	1.02	3.71
Mean of the five cultivars		49.9	50.1	1.00	4.12
Gluten isolate	Akteur – batch 1	48.7 ± 1.9	51.3 ± 2.1	0.95	4.63
	Akteur – batch 2	51.8^BCDE^ ± 2.1	48.2^BCD^± 1.9	1.07	4.66
	Carberry	53.8^ABC^ ± 2.2	46.2^DE^ ± 1.8	1.16	3.09
	Mv Magvas	52.8^BCD^ ± 2.1	47.2^CD^± 1.9	1.12	6.75
	Yitpi	53.7^ABC^ ± 2.1	46.3^DE^± 1.9	1.16	4.76
	Yumai-34	56.8^A^ ± 2.3	43.2^E^± 1.7	1.31	5.82
	Blend	54.4^AB^ ± 2.2	45.6^DE^± 1.8	1.20	4.58
Mean of the five cultivars		53.8	46.2	1.16	5.02
Gliadin isolate	Akteur – batch 1	–	–	–	4.98
	Akteur – batch 2	–	–	–	5.92
	Carberry	–	–	–	3.13
	Mv Magvas	–	–	–	6.18
	Yitpi	–	–	–	5.08
	Yumai-34	–	–	–	5.62
	Blend	–	–	–	5.96
Mean of the five cultivars		–	–	–	5.19
PWG-gliadin		–	–	–	5.34

*^*a*^ Within each column, the mean value of batch 1 Akteur samples marked with a plus sign is significantly different from the mean value of batch 2 Akteur samples (p < 0.05; one-way ANOVA). ^*b*^ Within each column, the measured value of the blend marked with an asterisk is significantly different from the calculated mean of the five cultivars (p < 0.05; one-sample t-test). ^*c*^ Within each column, mean values marked with different capital letters are significantly different (p < 0.05; factorial ANOVA, Fisher’s LSD).*

The composition of the different protein types within the monomeric and polymeric protein profiles determined by RP-HPLC are shown in Table 3. The proportion of different gluten protein types showed very similar values in the two flour batches and there were no significant differences in the proportion of total gliadin, ω1,2- and α-gliadin contents between the two batches. The gliadin/glutenin ratio of batch 1 was 1.7 and 1.6 in batch 2. The two batches of gluten isolates also showed great similarities and the difference was not significant in the ratio of α- and γ-gliadins (Table 3). The gliadin/glutenin ratio of batch 1 was 1.2 and 1.1 in batch 2. The distribution of different gliadin types was comparable in the two batches of gliadin isolates (Table 4). This demonstrates that the production of gliadin isolates yielded similar distributions of alcohol-soluble proteins.

**TABLE 3:** Proportion of gliadin, glutenin, and different gluten protein types in wheat flours and gluten isolates determined by RP-HPLC (RP-HPLC results are expressed as percentage of total extractable gluten proteins; all values are expressed on dry matter basis).

Sample		Parameter									
		Gliadins (%)^abc^	Glutenins (%)^abc^	Gliadin/Glutenin ratio	ω5 (%)^abc^	ω1,2 (%)^abc^	α (%)^abc^	γ (%)^abc^	ωb (%)^abc^	HMW- GS (%)^abc^	LMW- GS (%)^abc^
Flour	Akteur – batch 1	62.6 ± 0.4	37.4^+^ ±	1.7	6.1^+^ ± 0.0	6.4 ± 0.0	30.9 ± 0.2	19.3^+^ ± 0.1	1.7^+^ ± 0.0	11.3^+^ ± 0.1	24.3^+^ ± 0.2
	Akteur – batch 2	61.7^E^ ± 0.4	38.3^E^ ± 0.3	1.6	5.8^E^ ± 0.0	6.5^B^ ± 0.1	30.6^CD^ ± 0.2	18.8^I^ ± 0.1	1.5^F^ ± 0.0	11.8^B^ ± 0.1	25.0^CD^ ± 0.2
	Carberry	63.7^C^ ± 0.5	36.3^G^ ± 0.3	1.8	8.6^A^ ± 0.1	5.9^E^ ± 0.0	26.1^H^ ± 0.2	23.2^C^ ± 0.2	0.9^H^ ± 0.0	10.2^F^ ± 0.1	24.9^D^ ± 0.2
	Mv Magvas	65.9^AB^ ± 0.5	34.1^IJ^ ± 0.2	1.9	3.1^J^ ± 0.0	5.2^I^ ± 0.0	30.9^C^ ± 0.2	26.7^A^ ± 0.2	0.6^L^ ± 0.0	9.3^G^ ± 0.1	24.2^E^ ± 0.2
	Yitpi	65.3^B^ ± 0.5	34.7^H^ ± 0.2	1.9	6.7^C^ ± 0.1	6.2^D^ ± 0.0	29.2^E^ ± 0.2	23.2^C^ ± 0.2	0.7^K^ ± 0.0	10.9^E^ ± 0.1	23.1^G^ ± 0.2
	Yumai-34	65.6^AB^ ± 0.5	34.4^HI^ ± 0.2	1.9	5.4^G^ ± 0.0	6.9^A^ ± 0.1	32.9^A^ ± 0.2	20.3^G^ ± 0.1	0.9^I^ ± 0.0	11.2^D^ ± 0.1	22.4^H^ ± 0.2
	Blend	66.1^A^ ± 0.5	33.9^J^ ± 0.2	2.0	6.4^D^ ± 0.0	6.4^C^ ± 0.0	30.4^D^ ± 0.2	23.0^CD^ ± 0.2	0.8^J^ ± 0.0	10.3^F^ ± 0.1	22.7^H^ ± 0.2
Mean of the five cultivars		64.4*	35.6*	1.8	5.9*	6.1*	29.9	22.4*	0.9*	10.7*	23.9*
Gluten isolate	Akteur – batch 1	54.7^+^ ± 0.4	45.3^+^ ± 0.3	1.2	5.3^+^ ± 0.0	5.5^+^ ± 0.0	26.6 ± 0.2	17.2 ± 0.1	3.2^+^ ± 0.0	12.7^+^ ± 0.1	29.1^+^ ± 0.2
	Akteur – batch 2	53.4^I^ ± 0.4	46.6^A^ ± 0.3	1.1	4.7^I^ ± 0.0	5.3^H^ ± 0.0	26.4^GH^ ± 0.2	16.9^J^ ± 0.1	3.3^A^ ± 0.0	13.5^A^ ± 0.1	29.8^A^ ± 0.2
	Carberry	62.0^E^ ± 0.4	38.0^E^ ± 0.3	1.6	7.9^B^ ± 0.1	5.7^F^ ± 0.0	25.6^I^ ± 0.2	22.9^D^ ± 0.2	2.5^B^ ± 0.0	10.2^F^ ± 0.1	25.2^C^ ± 0.2
	Mv Magvas	57.6^H^ ± 0.4	42.4^B^ ± 0.3	1.4	2.9^K^ ± 0.0	4.6^J^ ± 0.0	26.3^GH^ ± 0.2	23.7^B^ ± 0.2	1.4^G^ ± 0.0	11.0^E^ ± 0.1	30.1^A^ ± 0.2
	Yitpi	59.7^G^ ± 0.4	40.3^C^ ± 0.3	1.5	5.5^F^ ± 0.0	5.4^G^ ± 0.0	26.5^G^ ± 0.2	22.2^E^ ± 0.2	2.2^C^ ± 0.0	12.0^B^ ± 0.1	26.1^B^ ± 0.2
	Yumai-34	62.8^D^ ± 0.4	37.2^F^ ± 0.3	1.7	5.2^H^ ± 0.0	6.3^D^ ± 0.0	31.9^B^ ± 0.2	19.4^H^ ± 0.1	1.7^E^ ± 0.0	11.6^C^ ± 0.1	23.8^F^ ± 0.2
	Blend	60.8^F^ ± 0.4	39.2^D^ ± 0.3	1.6	5.6^F^ ± 0.0	5.7^F^ ± 0.0	28.5^F^ ± 0.2	21.0^F^ ± 0.2	2.1^D^ ± 0.0	11.2^D^ ± 0.1	25.9^B^ ± 0.2
Mean of the five cultivars		59.1*	40.9*	1.5	5.3*	5.5*	27.3*	21.0	2.2*	11.7*	27.0*

**^*a*^Within each column, the mean value of batch 1 Akteur samples marked with a plus sign is significantly different from the mean value of batch 2 Akteur samples (p < 0.05; one-way ANOVA). ^*b*^Within each column, the measured value of the blend marked with an asterisk is significantly different from the calculated mean of the five cultivars (p < 0.05; one-sample t-test). ^*c*^Within each column, mean values marked with different capital letters are significantly different (p < 0.05; factorial ANOVA, Fisher’s LSD).**

**TABLE 4:** Proportion of different gliadin protein types in wheat flours, gluten and gliadin isolates determined by RP-HPLC (RP-HPLC results are expressed as percentage of total extractable gliadin proteins; all values are expressed on dry matter basis).

Sample		Parameter			
		ω5 (%)^abc^	ω1,2 (%)^abc^	α (%)^abc^	γ (%)^abc^
Flour	Akteur – batch 1	9.7 ± 0.1	10.2 ± 0.1	49.3 ± 0.4	30.8 ± 0.2
	Akteur – batch 2	9.5^F^ ± 0.1	10.5^D^ ± 0.1	49.6^D^ ± 0.4	30.5^M^ ± 0.2
	Carberry	13.4^B^ ± 0.1	9.2^I^ ± 0.1	41.0^L^ ± 0.3	36.4^E^ ± 0.3
	Mv Magvas	4.7^O^ ± 0.0	7.9^J^ ± 0.1	46.9^G^ ± 0.3	40.5^B^ ± 0.3
	Yitpi	10.2^D^ ± 0.1	9.6^G^ ± 0.1	44.7^IJ^ ± 0.3	35.5^G^ ± 0.3
	Yumai-34	8.3^K^ ± 0.1	10.5^D^ ± 0.1	50.2^C^ ± 0.4	31.0^L^ ± 0.2
	Blend	9.6^E^ ± 0.1	9.6^G^ ± 0.1	45.9^H^ ± 0.3	34.8^H^ ± 0.2
Mean of the five cultivars		9.2*	9.5	46.5	34.8
Gluten isolate	Akteur – batch 1	9.8 ± 0.1	10.0 ± 0.1	48.5 ± 0.3	31.4 ± 0.2
	Akteur – batch 2	8.9^I^ ± 0.1	10.0^F^ ± 0.1	49.4^D^ ± 0.4	31.7^K^ ± 0.2
	Carberry	12.7^C^ ± 0.1	9.1^I^ ± 0.1	41.2^L^ ± 0.3	36.9^D^ ± 0.3
	Mv Magvas	5.1^N^ ± 0.0	7.9^J^ ± 0.1	45.8^H^ ± 0.3	41.2^A^ ± 0.3
	Yitpi	9.3^G^ ± 0.1	9.1^I^ ± 0.1	44.4^J^ ± 0.3	37.2^D^ ± 0.3
	Yumai-34	8.3^JK^ ± 0.1	10.0^EF^ ± 0.1	50.8^B^ ± 0.4	30.9^L^ ± 0.2
	Blend	9.2^H^ ± 0.1	9.4^H^ ± 0.1	46.9^FG^ ± 0.3	34.5^H^ ± 0.2
Mean of the five cultivars		8.8*	9.2*	46.3*	35.6*
Gliadin isolate	Akteur – batch 1	9.9^+^ ± 0.1	10.1^+^ ± 0.1	49.1^+^ ± 0.3	30.9^+^ ± 0.2
	Akteur – batch 2	8.4^J^ ± 0.1	11.2^B^ ± 0.1	48.3^E^ ± 0.3	32.2^J^ ± 0.2
	Carberry	14.6^A^ ± 0.1	10.1^E^ ± 0.1	42.5^K^ ± 0.3	32.8^I^ ± 0.2
	Mv Magvas	5.3^M^ ± 0.0	9.3^H^ ± 0.1	47.3^FG^ ± 0.3	38.0^C^ ± 0.3
	Yitpi	9.3^G^ ± 0.1	9.6^G^ ± 0.1	45.2^I^ ± 0.3	36.0^F^ ± 0.
	Yumai-34	8.8^I^ ± 0.1	10.9^C^ ± 0.1	52.1^A^ ± 0.4	28.2^N^ ± 0.2
	Blend	7.9^L^ ± 0.1	12.0^A^ ± 0.1	47.4^F^ ± 0.3	32.6^I^ ± 0.2
Mean of the five cultivars		9.3*	10.2*	47.1	33.4*
PWG-gliadin		5.9 ± 0.3	7.2 ± 0.2	50.0 ± 0.4	36.9 ± 0.2

**^*a*^Within each column, the mean value of batch 1 Akteur samples marked with a plus sign is significantly different from the mean value of batch 2 Akteur samples (p < 0.05; one-way ANOVA). ^*b*^Within each column, the measured value of the blend marked with an asterisk is significantly different from the calculated mean of the five cultivars (p < 0.05; one-sample t-test). ^*c*^Within each column, mean values marked with different capital letters are significantly different (p < 0.05; factorial ANOVA, Fisher’s LSD).**

There was a high degree of similarity between the two batches of flour and gluten isolates in protein content and composition and this showed that the reproducibility of the production was similarly satisfactory. This was also confirmed by the ELISA results, as there were no significant differences between gliadin recoveries between the two batches of flours and gluten isolates (Table 5). Gliadin isolation appears to be more difficult due to a more complex process. Despite the variations in protein content and protein profile of the two batches of gliadin isolates, the ELISA results showed good similarity (Table 5). The amount of proteins obtained from gluten isolates along with the similarity of monomeric protein distribution indicated that the lower protein content and the presence or absence of higher molecular weight proteins on the SE-HPLC chromatograms has no effect on the ELISA results of gliadin fractions.

**TABLE 5:** Gliadin recovery in wheat flours, gluten and gliadin isolates using two different ELISA test kits (all values are expressed on dry matter basis; gliadin recoveries are calculated based on gliadin content measured by RP-HPLC).

Sample		Gliadin recovery (%)^abc^	
		
		ELISA kit A	ELISA kit B
Flour	Akteur – batch 1	187 ± 2	−
	Akteur – batch 2	163±BCD14	156±F29
	Carberry	183±AB36	179±EF24
	Mv Magvas	152±CDE13	157±F29
	Yitpi	169±ABCD21	190±EF18
	Yumai-34	165±BCD20	185±EF16
	Blend	150±DE19	183±EF25
Mean of the five cultivars		167	173
Gluten isolate	Akteur – batch 1	165 ± 40	−
	Akteur – batch 2	191±A17	215±CDE12
	Carberry	173±ABC26	281±AB116
	Mv Magvas	162±BCD18	206±CDEF10
	Yitpi	170±ABCD29	344±A38
	Yumai-34	176±AB14	215±CDE35
	Blend	182±AB15	260±BC81
Mean of the five cultivars		175	252
Gliadin isolate	Akteur – batch 1	123 ± 15	−
	Akteur – batch 2	132±E14	205±CDEF4
	Carberry	135±E10	187±EF8
	Mv Magvas	139±E8	223±BCDE11
	Yitpi	165±BCD27	201±CDEF12
	Yumai-34	132±E13	202±CDEF6
	Blend	180±AB11	244±BCD24
Mean of the five cultivars		140*	204
PWG-gliadin		125 ± 34	188 ± 55

**^*a*^Within each column, the mean value of batch 1 Akteur samples marked with a plus sign is significantly different from the mean value of batch 2 Akteur samples (p < 0.05; one-way ANOVA). ^*b*^Within each column, the measured value of the blend marked with an asterisk is significantly different from the calculated mean of the five cultivars (p < 0.05; one-sample t-test). ^*c*^Within each column, mean values marked with different capital letters are significantly different (p < 0.05; factorial ANOVA, Fisher’s LSD)*.*

In addition to reproducible production on laboratory scale, upscaling and reproducible production of larger amounts of material are also important for widespread use. In our previous study we managed to achieve this in the case of the five cultivars and the blend in flour form (Schall et al., 2020). In the case of isolates, the identification of sensitive points in laboratory production may help in upscaling their production.

### Comparison of the Different Gluten and Gliadin Isolates From Individual Cultivars and Their Blend

#### Comparison of the Protein Content of Flours, Gluten, and Gliadin Isolates

After testing the reproducibility of the production of flour and protein isolates from Akteur, the same methods were used for the other individual cultivars and for the blend of five cultivars. The crude protein content of the flours was in the range of 12.1–18.7% and the protein content of the blended flour was 15.4% which represented very well the calculated mean of the five cultivars (15.6%) and demonstrated the homogeneity of our flour blend (Table 1).

The crude protein content of gluten isolates from flours varied between 93.8 and 97.4% (Table 1). The lowest value belonged to Yitpi while Mv Magvas had the highest one. The high protein content means that comparatively pure gluten isolates could be extracted from each flour. The protein content of our gluten isolates from different flours was in a narrow range, but the variations may be due to the different separation behavior of cultivars during gluten processing (Marchetti et al., 2012). But the protein content of the isolates did not depend on the protein content of the flour. The amount of gluten proteins obtained from the proteins from different flours were in the range of 73.4–76.9%. Gluten yield may depend on the protein content of the flour (Van Der Borght et al., 2005), although no correlation was found between yield and protein content in the five samples we examined, meaning that the amount of gluten proteins was reached with similar potency for each sample (Table 1). The gluten isolated from the blended flour also had a high crude protein content of 95.5% while the mean of the five cultivars was 95.6%. The amount of gluten proteins relative to the proteins of the blend flour was 78.5% which was close, but a little higher than the mean of the five cultivars (75.6%) (Table 1). The crude protein content showed that the cultivars in the blend were affected to the same extent by isolation, however, the amount of proteins that could be obtained from the blend flour was higher compared to the individual cultivars. The difference was probably due to the production, as a difference was also observed in the yield of the two batches of Akteur. However, the protein content of the blend flour and gluten isolate was following the average of the five cultivars well so a detailed analysis of the protein profile is required.

The measured protein content of the gliadin isolates varied in the range of 72.7–101.9%, which was a much wider range than observed for the gluten isolates (Table 1). The protein content of PWG-gliadin was 92.8% (van Eckert et al., 2006), and from our samples, the Yitpi gliadin isolate had a higher protein content than PWG-gliadin. Despite the fact that we used exactly the same isolation procedure in each case, the crude protein contents of the gliadin isolates were significantly different. Additionally, it seems that the identified differences did not depend on the protein content of the flours or even the gluten isolates. In case of gluten isolates, it can be assumed that the differences between the samples depended on cultivars. This cannot be clearly stated for the gliadin isolates, because as shown in the investigation of reproducible production, such differences may occur between up to two parallel isolations and the causes of this phenomenon must be revealed. Less pure gliadin isolates thus raise the question if lower protein contents would cause changes in the protein profiles compared to flours and gluten isolates, and consequently in ELISA response. The amount of gliadin proteins obtained from different gluten isolates was between 28.8 and 58.2% (Table 1). Gliadin/glutenin ratios based on the weight of the isolated gliadins and glutenins were in the range from 0.5 to 1.4. Such variability in extractable gliadin content and gliadin/glutenin ratios in gluten proteins among cultivars may occur, but this degree of variation did not occur in the production of flour from these cultivars (Hajas et al., 2018; Schall et al., 2020). It is therefore necessary to examine how the differences are affected by the quality of the cultivars and what the effect of isolation is. The protein content of the blend gliadin isolate was 79.6% and the calculated mean of the five cultivars was 84.2%, which was a higher (but still not significant) difference than in case of the blend gluten isolate (Table 1). The amount of gliadin obtained from the gluten isolate was 28.8% and the mean of the five cultivars was 43.3% meaning that the theoretical protein content available in the blend gliadin isolate could not be extracted and loss of material could be problematic, as the profile may also change compared to flour. Just as gluten production is affected by different protein interactions, the separation of gliadins and glutenins can also be determined by the different types of proteins in the material. The yield obtained for the blend was lower than in case of any cultivar, and we did not obtain such a difference in the parallel yields of Akteur, so this is difficult to explain with the uncertainty of the isolation method. It is conceivable that glutenins from different cultivars in the blend are able to aggregate strongly, thus negatively affecting the yield of gliadin.

Protein yields in the gluten isolates with a high protein content were well representative of flour values, while the protein contents and the amount of extracted proteins in gliadin isolates were slightly distorted because in some cases gliadin isolates with lower protein content were obtained than for gluten isolates. Protein isolates with high protein content were obtained with our isolation method that are comparable to commercially available materials and PWG-gliadin (van Eckert et al., 2006; Schwalb et al., 2011). Schalk and colleagues dealt with the development of a strategy for the isolation of protein fractions and types from different species, in which the crude protein contents of the different isolates were similar to our isolates (Schalk et al., 2017). However, protein content and yield alone are not sufficiently informative, because a comparative study has already shown that the composition of gliadin isolates is highly dependent on production (Schwalb et al., 2011). In the following paragraphs, the protein composition of the isolates is described to evaluate their identity compared to the gluten proteins of the source flour.

#### Comparison of the SE-HPLC Protein Profiles of Flours, Gluten, and Gliadin Isolates

**FIGURE 2:**
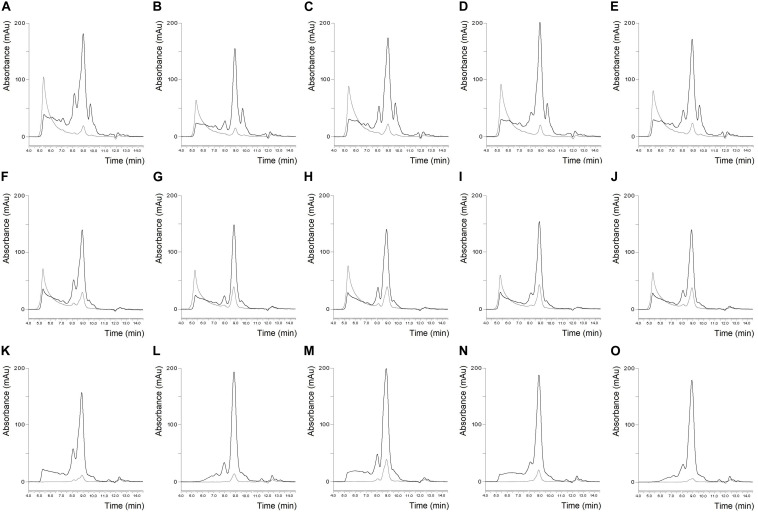
Protein profile of Carberry flour **(A)**, Mv Magvas flour **(B)**, Yitpi flour **(C)**, Yumai-34 flour **(D)**, Blend flour **(E)**, Carberry gluten isolate **(F)**, Mv Magvas gluten isolate **(G)**, Yitpi gluten isolate **(H)**, Yumai-34 gluten isolate **(I)**, Blend gluten isolate **(J)**, Carberry gliadin isolate **(K)**, Mv Magvas gliadin isolate **(L)**, Yitpi gliadin isolate **(M)**, Yumai-34 gliadin isolate **(N)** and Blend gliadin isolate **(O)** determined by SE-HPLC (black line: soluble fraction, gray line: insoluble fraction).

The SE-HPLC protein profiles of the six flours and their isolates are shown in Figures 1, 2. Naturally, the sizes of the albumin/globulin peaks were smaller in the gluten isolates compared to the flours, but a small residue was observed in the different gluten samples with varying degrees. The effectiveness of removing water and salt-soluble proteins may depend on cultivars and small variations during isolation. In all six cases, a similar protein composition was seen in the distribution of polymeric and monomeric proteins for gluten isolates compared to the flours which were specific to the cultivars. The greatest change was identified in the higher molecular weight regions of the soluble fractions of all gluten isolates, where the protein profile was slightly modified. There was also a change in monomeric proteins in the insoluble fraction, as their amount increased compared to flours in all cases. Similar findings were made in the blend gluten isolate as in the individual cultivars because the amount of albumins/globulins decreased. A slight change in the size distribution of higher molecular weight proteins could also be observed, as the increase in the amount of monomeric proteins in the insoluble fraction. However, the protein distribution characteristics of the blend flour were also reflected in the gluten isolate. In flour samples, the proportion of monomeric proteins was in the range of 46.9–51.8%, while the values of polymeric proteins were between 48.2 and 53.1% (Table 2). There was an increase in the monomeric protein content of the gluten isolates (51.8–56.8%), compared to flour and the extent of change was not the same for all samples, because it was smaller in the case of Carberry than in the other cultivars. The increase due to isolation in the proportion of monomeric proteins in the gluten isolates was also reflected in changes in the monomeric/polymeric protein ratio (Table 2). The increase in the proportion of monomeric proteins also occurred in the blend gluten isolate compared to flour. So the change in the ratio of monomeric and polymeric proteins due to isolation was similar in the blend and the individual cultivars. The monomeric protein content of the blend gluten isolate was 54.4% while the mean of the five cultivars was 53.8%. This similarity was also observed in the amount of polymeric proteins as the blend gluten isolate had 45.6% while the mean was 46.2%. So it represented the average of the five individual gluten isolates. Overall, there were no major differences in the gluten protein composition between gluten isolates and flours, but any change in proportions could be problematic because the flour-specific ratio would be affected, which could cause further uncertainty in methods where only alcohol-soluble proteins are determined.

The size distribution of proteins in the gliadin isolates is shown in Figures 1, 2. As expected, the peak size of albumin/globulin proteins decreased further in gliadin isolates compared to flours and gluten isolates. In most cases albumins/globulins were completely missing, but small residues could be observed, for example, in the case of Yitpi and Mv Magvas. The peaks of monomeric proteins appearing in the soluble fractions (between 7.5 and 9.5 min) followed the pattern observed in the flours and gluten isolates for each sample. The ratio of the two peaks of the monomeric proteins appearing in the gliadin isolates showed higher values than in flours but showed similarities with gluten isolates. It means that the production of gluten isolates had a greater effect on the distribution of these proteins, while the production of the gliadin isolate had a smaller impact. The smallest change of the two peaks in monomeric proteins was observed in the case of Carberry, as the gluten and gliadin isolates showed only a slight increase compared to flour. As mentioned above, the peak of monomeric proteins in the insoluble fraction increased in the gluten isolates compared to flours, while it decreased in most of the gliadin isolates compared to gluten, making it more comparable to flours. As expected, the higher molecular weight proteins were reduced in gliadin isolates, and completely disappeared in the insoluble fraction for each sample. Although we expected the lack of these proteins in the soluble fraction, they did appear in Carberry, Yitpi, and Yumai-34 while they could not be detected in Akteur and Mv Magvas gliadin isolates. However, it was shown during the examination of reproducible production that the presence or absence of these proteins was somehow influenced by the conditions of production or the sample preparation of the method, not just the cultivars. Similar to most of the individual cultivars, the albumin/globulin peak completely disappeared in the blend gliadin isolate. There was an increase in the ratio of the two peaks of the monomeric proteins in the soluble fractions, but the degree of change was higher compared to the gluten isolate and flour as for the individual cultivars (Table 2). The ratio of the blend gliadin isolate was 5.96 while the mean of the five cultivars was 5.19 which showed a slight distortion compared to the theoretical ratio (Table 2). This points to uncertainties of production and its reason must be explored in order to standardize the production of RM, and to reduce random errors originating from production. The amount of monomeric proteins in the insoluble fraction decreased compared to gluten isolates but the reduction was higher than in other gliadin isolates (Figures 1, 2). Another important finding is that the higher molecular weight proteins were also missing in the soluble fraction of the blend gliadin isolate.

**FIGURE 3:**
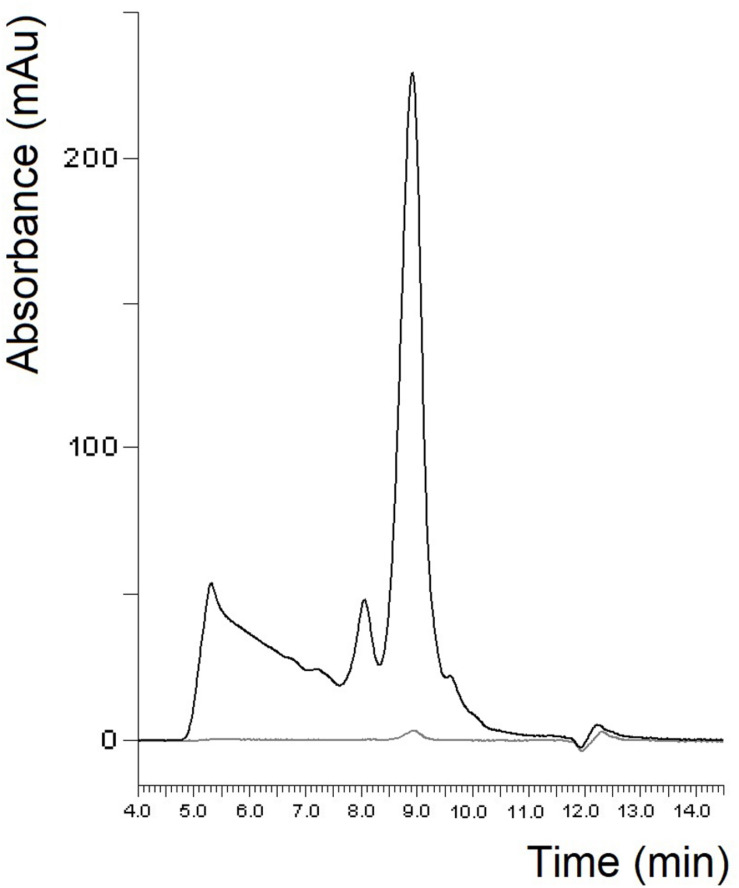
Protein profile of PWG-gliadin determined by SE-HPLC (black line: soluble fraction, gray line: insoluble fraction).

In case of gliadin isolates, it seems reasonable to compare our results with the well-known PWG-gliadin. As in some of our gliadin isolates, PWG-gliadin also had a small albumin/globulin peak (Figure 3; van Eckert et al., 2006). The amount of monomeric proteins in the insoluble fraction was much lower than in our study. As expected and similar to our samples, two peaks appeared in the monomeric proteins of the soluble fraction, with a ratio of 5.34 (Table 2). Some of our individual cultivars considerably differed from this value, but the value of our blend gliadin isolate was very close to it. This may mean that a mixture actually represents a number of wheat cultivars, but it may not be necessary to include a large number of cultivars (28 in case of PWG-gliadin), but a smaller number of carefully selected varieties which can facilitate the production of RM. There were no higher molecular weight proteins in the insoluble fraction while they appeared in the soluble fractions of PWG-gliadin (Figure 3). In the article on the characterization of PWG-gliadin (van Eckert et al., 2006), these proteins were called oligomeric gliadin and based on our results, the presence of these proteins may be matrix- and/or production-dependent.

#### Comparison of the RP-HPLC Protein Profiles of Flours, Glutens, and Gliadin Isolates

**FIGURE 4:**
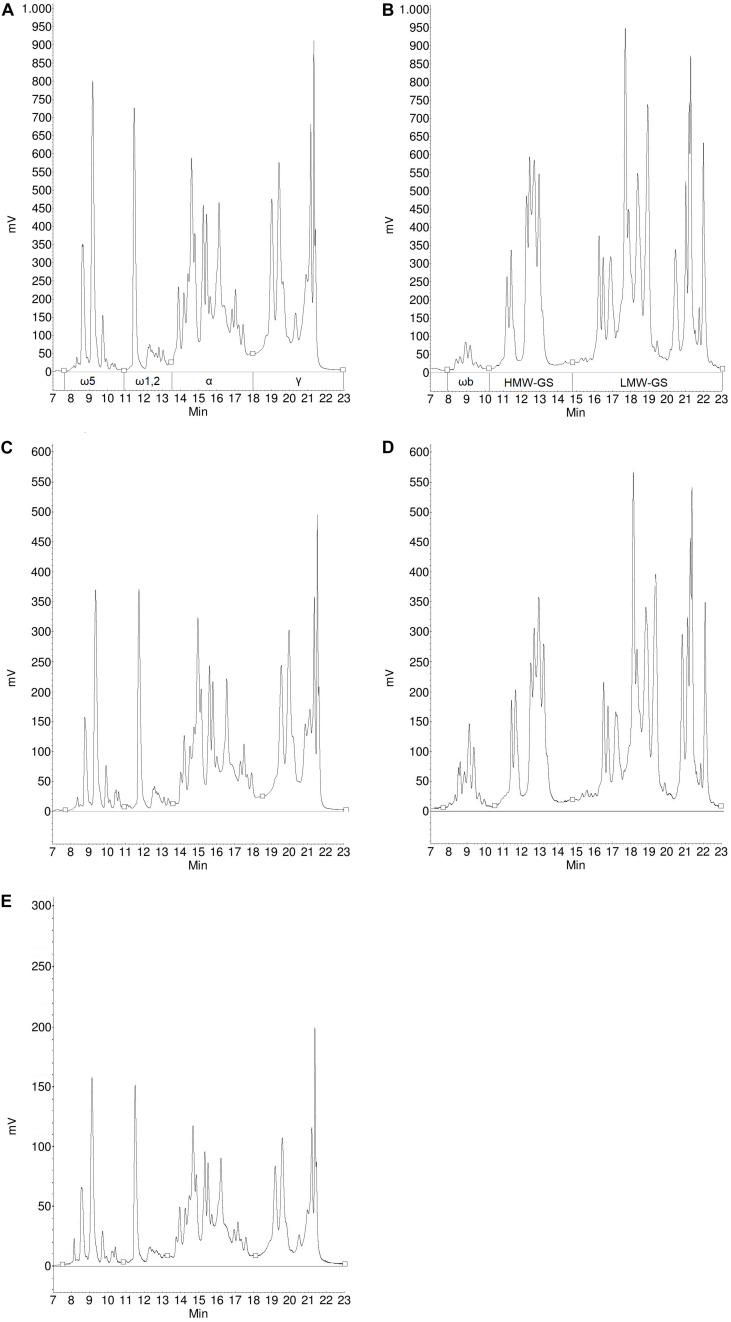
Protein profile of gliadins **(A)** and glutenins **(B)** from Carberry flour, gliadins **(C)** and glutenins **(D)** from Carberry gluten isolate and gliadins **(E)** from Carberry gliadin isolate determined by RP-HPLC.

**FIGURE 5:**
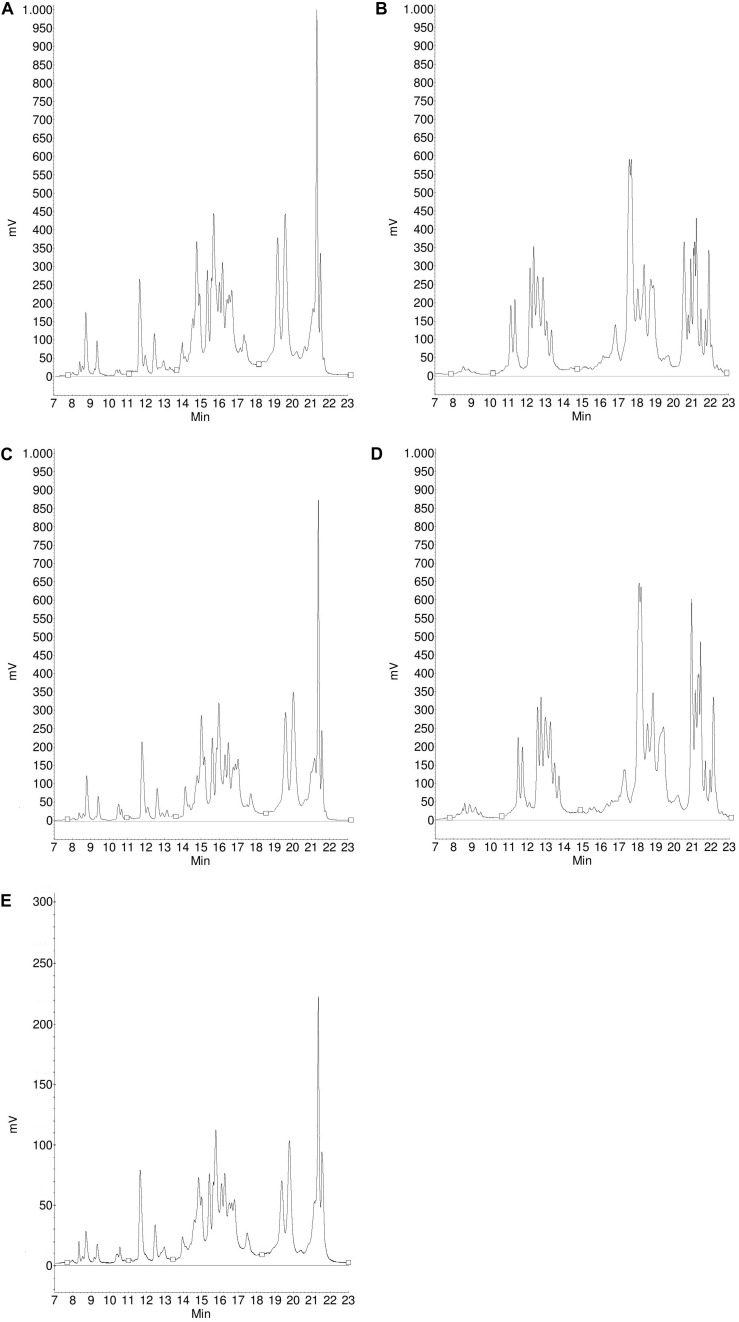
Protein profile of gliadins **(A)** and glutenins **(B)** from Mv Magvas flour, gliadins **(C)** and glutenins **(D)** from Mv Magvas gluten isolate and gliadins **(E)** from Mv Magvas gliadin isolate determined by RP-HPLC.

The composition of different gluten proteins in flours and gluten isolates separated by RP-HPLC for more detailed examination is shown in Table 3. Based on the RP-HPLC chromatograms, the protein composition showed greater similarity between flours and isolates for each sample than in the SE-HPLC chromatograms, so only the RP-HPLC chromatograms for Carberry and Mv Magvas are shown to demonstrate the results in Figures 4, 5. There was a slight decrease in the percentage distribution of ω5- and ω1,2-gliadins in gluten isolates compared to flour, but the extent of change was similar for all samples (Table 3). Washing with tap water to remove residual starch in gluten production could cause a slight decrease in the amount of ω-gliadins because these types of proteins are slightly soluble in water. The α- and γ-gliadins also showed a decrease in gluten isolates compared to flours, but the rate of change was not the same. In case of Carberry, the changes were negligible, while Mv Magvas showed the greatest extent both in α- and γ-gliadins. Consequently, the total gliadin content also decreased in the gluten isolates to a different degree relative to the flours, and the slightest change occurred in Carberry. A moderate decrease could be detected in the proportion of different gliadin proteins for blend gluten isolates compared to flours. However, the values of the blend gluten isolate showed great similarity with the average of the five gluten isolates, so it was representative of the five gluten isolates. In accordance with the change in the amount of gliadins, the proportion of glutenins in the gluten isolates was increased compared to the flours. The extent of change was similar for each sample in ωb-gliadins and HMW-GS. While in LMW-GS, the rate of increase was different in the cultivars, the highest change was in Mv Magvas and the lowest was in Carberry, similar to α- and γ-gliadins. In the blend gluten isolate, the proportion of glutenin proteins increased compared to the blend flour to a similar extent as in the individual cultivars. It can also be seen that the values of the blend gluten isolate were very close to the mean of the five isolates from the individual cultivars.

Based on analysis of variance, the variability between cultivars was higher (67% in ω5-, 42% in ω1,2-, 40% in α-, 58% in γ-gliadins and 43% in HMW-GS) than the effect of isolation (19% in ω5-, 38% in ω1,2-, 33% in α-, 24% in γ-gliadins and 31% in HMW-GS) in all types of gliadins and also in HMW-GS. This also means that the potential loss of ω-gliadins during isolation is expected to cause less error than the genetic variability. The differences originating from the cultivars should be reduced, which was achieved not only with the blend flour but also with the blend gluten isolate, because the gliadin and HMW-GS contents were close to the mean of the five cultivars. However, the isolation had a higher effect (56% in ωb-gliadins and 42% in LMW-GS) than variability between cultivars (25% in ωb-gliadins and 2% in LMW-GS) in case of ωb-gliadins and LMW-GS.

The composition of different gliadin proteins in flours, gluten and gliadin isolates separated by RP-HPLC are shown in Table 4. The chromatograms of the Carberry and Mv Magvas gliadin isolates are shown in Figures 4, 5. As in the case of gluten isolates, the effect of isolation was less noticeable in the distribution of ω5- and ω1,2-gliadins, because gliadin isolates had similar values to flours and gluten isolates. In ω5-gliadins, the direction of change was also positive and negative depending on the sample compared to glutens and flours while in the case of ω1,2-gliadins, the values of gliadin isolates were higher than in flours and gluten isolates. The α-gliadins also showed very small changes in the distribution compared to flours and gluten isolates. The greatest change due to isolation was found in γ-gliadins. While gluten isolates had a very similar value to flours in every cultivar, the γ-gliadin proportions of gliadin isolates varied and not to the same extent. Interestingly, a high difference occurred in Carberry despite the fact that the gluten isolate showed the best similarity to the flour. The variance analysis with the results of the gliadin isolates also showed that the variability between cultivars had a greater effect (79% in ω5-, 41% in ω1,2-, 71% in α-, and 60% in γ-gliadins) on the distribution of different proteins than the isolation (0% in ω5-, 32% in ω1,2-, 7% in α-, and 17% in γ-gliadins). The change in the blend gliadin isolate was small compared to the gluten isolate and flour and the difference between the values of the blend isolate and the mean of the five gliadin isolates was small. This means that the blend gliadin isolate adequately represented the mean of the five cultivars. Consequently, the isolation did not alter the homogeneity of the proteins and each gliadin type was isolated with the same efficiency. This answered the question above related to protein content and showed that we did not lose information with reduced protein recovery, because the gluten protein composition was very similar in flour and isolates.

In the case of PWG-gliadin, it was found that its composition was very similar to the distribution of gliadin proteins in the source flour (van Eckert et al., 2006). Our gliadin isolates typically had higher ω5- and ω1,2-gliadin contents and lower α- and γ-gliadin contents than PWG-gliadin (Table 4). Gluten and gliadin isolates with high protein content are available, but their protein composition is not similar or probably altered compared to the composition of wheat flour (Schwalb et al., 2011). Despite the minor changes observed in the composition during isolation, our isolates – both gluten and gliadins – were similar to the source flour, as well as to PWG-gliadin.

#### Comparison of the Gliadin Recovery Values of Flours, Gluten, and Gliadin Isolates Measured With ELISA Tests

In the next step we investigated whether the slight and different modifications described above were apparent in the ELISA results. Gliadin recovery values (Table 5) of ELISA measurements were calculated relative to the total gliadin content measured by RP-HPLC. For each sample and both ELISAs, the recovery values were above 100%. Lexhaller et al. (2016) noted that both G12 and R5 ELISA kits overestimated the prolamin content relative to RP-HPLC results in wheat. Despite high recovery values, it is informative to compare the results obtained with different samples and methods. The recovery values for gluten isolates obtained by ELISA method A showed good similarity to flours and there were no significant differences between the two. The highest difference between flour and the gluten isolate occurred in cultivar Akteur with an increase of about 30%. The blend had a higher change in the isolate compared to most cultivars, with about the same extent as in Akteur. However, the recovery value of the blend gluten isolate was closer to the mean of the five gluten isolates than in the flour blend. Since the protein profiles of gluten isolates were similar to flours based on both SE- and RP-HPLC results, similarities in ELISA results were expected.

In case of ELISA method B, there was a much greater increase in the recovery values of gluten isolates compared to flour (Table 5) than with ELISA method A. The rate of change varied between cultivars; the smallest increase was in Yumai-34, while the highest was in Yitpi. The recovery value of the blend gluten isolate was higher compared to flour, but the blend was comparably close to the mean of the five gluten isolates compared to the blend flour and the mean of the five flours. This is only possible if the blend contains the five cultivars homogeneously in the flour and in the gluten isolate. Considering the results of gluten protein composition, no protein types or even the total gliadin content could be identified that would be associated with the increase in ELISA results in some gluten isolates.

Increases were mainly observed in the recovery values of gluten isolates compared to flour, while the values of gliadin isolates decreased in all cases but not to the same extent in case of method A. The lowest change was observed in Yitpi, while the highest was in Carberry. Interestingly, the value of the blend increased compared to flour in contrast to individual cultivars and its value was very similar to the blend gluten isolate. The recovery value of the blend gliadin isolate differed quite substantially from the mean of the five cultivars. Consequently, it was more affected by isolation than by cultivars. The recovery value of PWG-gliadin was lower than our gliadin samples (Table 5), but the mean value of the five cultivars did not show a very high difference from PWG-gliadin.

Interestingly, in case of method B, the recovery values of the gliadin isolates showed better similarity to the flours than the gluten isolates (Table 5). In the blend gliadin isolate (similarly to the results of method A) there was an increase compared to flour and its value was more similar to the gluten isolate than the flour. The value of the blend did not differ significantly from the mean of the five cultivars. A lower recovery value was determined for PWG-gliadin than for our samples, except Carberry and there was also a marked difference between the value of our blend gliadin isolate and PWG-gliadin (Table 5).

The results obtained with ELISA methods show variation between materials of different formats and compositions (Schwalb et al., 2011; Lexhaller et al., 2016). In addition, the reactivity of various antibodies can be different, which causes serious uncertainty in the measurement results (Schwalb et al., 2011; Lexhaller et al., 2017). It is difficult to determine which factor is most relevant for the deviation of the results and their relevance for the production of the RM. Protein composition results showed that the isolation had a minimal effect on protein distribution. The results obtained with both ELISA methods were assessed by analysis of variance. The isolation had a significant effect on the results of both ELISA methods because it contributed 28% to the deviation of the results in case of method A and 36% in case of method B. The variation between cultivars also affected the results, but to a lower extent than isolation. The degree of interaction between isolation and genetic variability was 29% for method A, while it was 20% for method B. Genetic variability contributed by an additional 11% to the deviation of the measured values in case of method B. Furthermore, the measurement uncertainty in both methods approximated but did not exceed the effect of isolation and genetic variability. This points to the importance of carefully choosing a RM to minimize these effects. One argument for using flour is that there was no significant difference in the values of the two methods for any of the samples, but the difference between the cultivars could be reduced by the use of the blend, since there was no significant difference between its values and the mean of the five cultivars.

## Conclusion

We have shown that the isolation of gluten and gliadin proteins from wheat flours has a slight effect on the amount and composition of proteins, which partially depends on the cultivar. However genetic variability still causes higher uncertainty in protein composition than isolation. Obviously, due to the more complex production of gliadins, there is a higher probability of error, and we tried to investigate the causes of it in our study. Immunoanalytical results showed higher effects of isolation on the results and they seem to be method dependent. We demonstrated that the directions and extents of changes are not in direct relation to the other protein properties. Similarly, to our previous results with flours (Schall et al., 2020), here we also confirmed experimentally that the blend is the most appropriate solution to compensate for the effects of genetic variability. As an overall conclusion of our work, we demonstrated first with analytical experiments that similar results can be obtained with isolates than with basic flours. The blends can partly compensate for the effects of genetic and environmental variability and the source and the extent of analytical uncertainty are similar (but not the same) in all investigated materials. In the gliadin isolation process, there is a higher chance of uncertainty that can affect the analytical results. Exploring the sources of these errors, evaluating the results of long-term stability studies, and clarifying the specific analytical goals are necessary to select the suitable form or forms of a widely accepted RM. One limitation of the study is that we have only completed the work for wheat so far, but our experience will now enable us to transfer our findings efficiently to producing RM for rye and barley, as well, and thus provide a comprehensive gluten RM.

## Data Availability Statement

All datasets generated for this study are included in the article/supplementary material.

## Author Contributions

ST, PK, KS, and RS contributed to the conception and design of the study. ES, KS, ZB, and KT contributed to performing the experiments. ES organized the database and performed the statistical analysis. ES and ST evaluated the results and wrote the first draft of the manuscript. All authors contributed to the article and approved the submitted version.

## Conflict of Interest

PK was employed by the company Biotask AG (Esslingen am Neckar, Germany). The remaining authors declare that the research was conducted in the absence of any commercial or financial relationships that could be construed as a potential conflict of interest.

## Abbreviations

ANOVA: analysis of variance
ELISA: enzyme-linked immunosorbent assay
HMW-GS: high-molecular-weight glutenin subunits
HPLC: high-performance liquid chromatography
LC-MS: liquid chromatography mass spectrometry
LMW-GS: low-molecular-weight glutenin subunits
LSD: least significant difference
RM: reference material
RP: reversed-phase
SE: size-exclusion
TFA: trifluoroacetic acid
ω b: glutenin-bound ω-gliadin.
